# When ownership stakes pull in different directions: How CGHRM practices shape the ethical implications of employee stock ownership level

**DOI:** 10.3389/fpsyg.2026.1974402

**Published:** 2026-10-28

**Authors:** Xinyi Yin, Zhaoyu Wang, Can Zhang

**Affiliations:** 1 Overseas Chinese College, Capital University of Economics and Business, Beijing, China; 2 School of Engineering and Technology, Southwest University, Chongqing, China; 3 School of Economics and Management, Beijing Jiaotong University, Beijing, China

**Keywords:** bottom-line mentality, employee stock ownership level, felt responsibility, perceived CGHRM practices, unethical pro-organizational behavior

## Abstract

**Introduction:**

Employee stock ownership is widely used to align employee and organizational interests, yet its ethical implications across different ownership levels remain insufficiently understood. Drawing on goal-framing theory, we develop a conditional dual-path model linking employee stock ownership level to unethical pro-organizational behavior through felt responsibility and bottom-line mentality, with perceived corporate green human resource management (CGHRM) practices moderating these pathways.

**Methods:**

We tested the proposed model using three-wave, multisource data collected from 303 employee–coworker dyads in three Chinese firms.

**Results:**

Perceived CGHRM practices strengthened the positive association between employee stock ownership level and felt responsibility but weakened the positive association between employee stock ownership level and bottom-line mentality. Accordingly, the negative indirect association between employee stock ownership level and unethical pro-organizational behavior through felt responsibility was stronger when perceived CGHRM practices were higher, whereas the positive indirect association through bottom-line mentality was stronger when perceived CGHRM practices were lower.

**Discussion:**

This study extends the employee stock ownership literature to the domain of employee ethical behavior by identifying two contrasting psychological pathways linking ownership level to unethical pro-organizational behavior. It also advances a more contextualized understanding of the ethical implications of employee ownership arrangements by highlighting the moderating role of perceived CGHRM practices.

## Introduction

Employee stock ownership refers to an organizational arrangement through which employees acquire equity interests in their employing firms and thereby assume ownership rights in addition to their employee roles (Long, 1978; Pierce et al., 1991). By enabling employees to share in firms’ value appreciation and operating outcomes, employee stock ownership links employees’ economic interests with organizational performance and positions them as direct stakeholders in organizational outcomes (Klein, 1987; Kim et al., 2025; Pendleton and Robinson, 2010). Existing research has primarily focused on the positive consequences of employee stock ownership, demonstrating that it enhances organizational identification and commitment, improves work attitudes, and contributes to individual- and organization-level performance outcomes (Kim and Han, 2019; O’Boyle et al., 2016; Rodríguez-Oramas et al., 2022; Weissenböck et al., 2024). Accordingly, employee stock ownership has long been viewed as an important organizational mechanism for aligning employee and organizational interests, sharing organizational gains, and fostering employee involvement (Pierce et al., 1991; Pendleton and Robinson, 2023). Nevertheless, prior research has also identified potential drawbacks of employee ownership, including free-riding that may weaken its incentive effects and managerial entrenchment that may undermine corporate governance (Aubert et al., 2014; Kim and Ouimet, 2014). Beyond these organizational-level concerns, even among employee-owners, the extent of ownership stakes can vary considerably, and whether such differences are associated with distinct employee outcomes remains unclear.

A stronger alignment between employee and organizational interests does not necessarily mean that employees will pursue organizational interests in ethically appropriate ways. Unethical pro-organizational behavior refers to behaviors intended to benefit the organization or its members but that violate broader ethical standards, social norms, or legal requirements (Umphress et al., 2010; Umphress and Bingham, 2011; Wang et al., 2021). Prior research has primarily emphasized the roles of organizational identification, positive reciprocity, and pro-organizational motives in motivating unethical pro-organizational behavior (Mo et al., 2023; Umphress et al., 2010; Umphress and Bingham, 2011). However, recent research further suggests that unethical pro-organizational behavior does not stem exclusively from motives to benefit the organization; self-interested motives can also serve as an important source of such behaviors (Mo et al., 2023; Steele et al., 2024; Xu et al., 2022).

This perspective is particularly important for understanding the potential ethical risks associated with employee stock ownership. Unlike pro-organizational orientations rooted solely in affective attachment or organizational identification, employee ownership directly links organizational performance to employees’ own economic interests (Long, 1978; Pierce et al., 1991). As ownership stakes increase, organizational performance becomes not only consequential for the organization itself but also increasingly tied to the economic value of employees’ personal equity holdings. Thus, efforts to enhance organizational performance may simultaneously serve organizational interests and employees’ own economic interests, creating potential tension between helping the organization and adhering to ethical standards. If overlooked, employee ownership may unintentionally encourage behaviors that generate short-term organizational benefits while exposing firms to compliance, reputational, and stakeholder-related risks (Mo et al., 2023; Umphress and Bingham, 2011). Therefore, an important question remains: among employee-owners, how and under what conditions do differences in ownership levels relate to unethical pro-organizational behavior?

To address this question, we develop our theoretical model based on goal-framing theory. This theory suggests that individuals’ judgments and behaviors are shaped by different goal frames, and that contextual factors influence the relative salience of these goal frames (Lindenberg and Foss, 2011; Yin et al., 2025). Specifically, the gain goal frame emphasizes the maintenance and improvement of personal resources, whereas the normative goal frame emphasizes acting in accordance with collective goals and standards of appropriateness (Lindenberg and Foss, 2011). In the context of employee stock ownership, higher ownership stakes make organizational performance simultaneously relevant to employees’ economic interests and their responsibilities as organizational members. As a result, greater ownership stakes may alter the relative salience of different goal frames and give rise to divergent psychological pathways. When the normative goal frame becomes more salient, employees are more likely to translate their connection with organizational outcomes into a psychological state characterized by taking personal responsibility for their work and its consequences, namely felt responsibility (Pearce and Gregersen, 1991; Fuller et al., 2006), thereby reducing the likelihood of crossing ethical boundaries in the name of organizational interests. In contrast, when the gain goal frame becomes more salient, organizational performance receives greater priority because of its connection with employees’ personal benefits, making employees more likely to develop a bottom-line mentality, which prioritizes bottom-line outcomes over other goals (Greenbaum et al., 2012, 2023), thereby making them more likely to rationalize unethical pro-organizational behavior.

Goal-framing theory further suggests that the relative salience of different goal frames is not fixed but is shaped by individuals’ contextual environments and external cues (Lindenberg and Foss, 2011). Accordingly, the psychological responses elicited by employee stock ownership depend not only on ownership levels themselves but also on how the organizational context shapes the goals that employees attend to. Common good human resource management (CGHRM) emphasizes that organizations should move beyond a sole focus on economic performance in their human resource management practices and incorporate the well-being of employees, other stakeholders, and broader societal interests into their core considerations (Aust et al., 2020; Lu et al., 2025; Pham et al., 2023). Therefore, CGHRM may influence the relative salience of different goal frames when employees respond to employee ownership arrangements. When employees perceive higher levels of CGHRM, considerations associated with the normative goal frame are more likely to serve as important bases for behavioral judgments, making greater ownership stakes more likely to enhance felt responsibility and reduce employees’ likelihood of engaging in unethical pro-organizational behavior. In contrast, when employees perceive lower levels of CGHRM, considerations associated with the gain goal frame are more likely to shape responses to employee ownership arrangements, making greater ownership stakes more likely to strengthen bottom-line mentality and increase employees’ likelihood of engaging in unethical pro-organizational behavior. Figure 1 presents our theoretical model.

**Figure 1:**
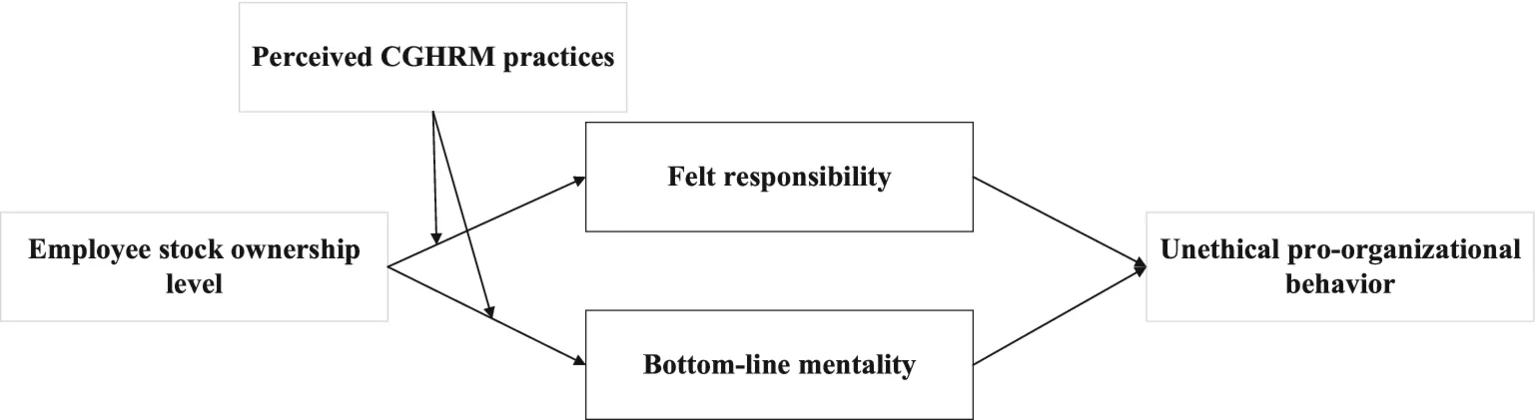
Theoretical model.

We aim to make three contributions. First, existing research has primarily focused on the positive effects of employee stock ownership on employee attitudes, employee behaviors, and organizational performance (Dasilas, 2024; Kim and Han, 2019; Kim et al., 2025; O’Boyle et al., 2016; Pendleton and Robinson, 2023), while paying limited attention to whether differences in ownership levels among employee-owners may influence employees’ ethical behaviors when pursuing organizational interests. By introducing unethical pro-organizational behavior into the employee stock ownership literature, we reveal the potential ethical risks associated with employee–organization interest alignment and extend understanding of the consequences of employee stock ownership. Second, drawing on goal-framing theory, we identify the dual psychological pathways through which employee stock ownership is associated with unethical pro-organizational behavior, namely felt responsibility and bottom-line mentality. We show that employee stock ownership does not necessarily produce a uniform ethical consequence; rather, higher ownership stakes may reduce unethical pro-organizational behavior through enhanced felt responsibility while increasing unethical pro-organizational behavior through heightened bottom-line mentality. Third, prior research on common good human resource management (CGHRM) has primarily examined its positive effects on meaningfulness at work, thriving, ethical behavior, and prosocial behavior (Aust et al., 2020; Lu et al., 2023; Lu et al., 2025; Pham et al., 2023). We further introduce CGHRM into the employee stock ownership context and propose that CGHRM, as an organizational contextual factor, can shape the relationship between employee stock ownership and unethical pro-organizational behavior.

## Theory and hypotheses

### Goal-framing theory

Goal-framing theory examines how individuals determine the basis for action when multiple goals coexist. It suggests that individuals’ behaviors are not continuously driven by a single goal but depend on which goal frame becomes more salient in a given situation (Lindenberg, 2001; Lindenberg and Foss, 2011). The theory further proposes that goal frames influence not only the information individuals attend to but also how they evaluate behavioral choices and the value of potential outcomes (Lindenberg and Steg, 2007; Lindenberg and Foss, 2011). Specifically, the gain goal frame directs individuals’ attention toward the acquisition, maintenance, and improvement of resources, emphasizing the implications of actions for personal interests, whereas the normative goal frame focuses on whether behaviors conform to social norms, collective goals, and standards of appropriateness, highlighting considerations of responsibility and obligation (Lindenberg and Foss, 2011). Because different goal frames rely on distinct evaluative criteria, the same situation may be interpreted differently and consequently lead to different behavioral choices (Lindenberg and Steg, 2007). Importantly, goal-framing theory suggests that the relative salience of different goal frames is not fixed but can be influenced by environmental conditions and contextual cues (Lindenberg, 2001; Lindenberg and Foss, 2011). Therefore, this theory provides an important foundation for explaining why the same contextual factor in organizational settings may generate different psychological processes and behavioral outcomes (Lindenberg and Foss, 2011; Yang et al., 2021; Yin et al., 2025).

Goal-framing theory provides an appropriate framework for explaining how employee stock ownership influences unethical pro-organizational behavior. In our model, employee stock ownership represents an organizational arrangement that may simultaneously activate the gain goal frame and the normative goal frame. Specifically, employees may interpret employee stock ownership as an incentive arrangement linking their personal economic returns to organizational performance, thereby activating the gain goal frame. Alternatively, they may interpret ownership as an arrangement through which the organization grants them an ownership role and expects them to assume broader responsibilities, thereby activating the normative goal frame (Pierce et al., 1991; McConville et al., 2016). CGHRM, as an HRM orientation emphasizing the well-being of employees, stakeholders, and broader society, influences the relative salience of different goal frames when employees respond to employee stock ownership arrangements (Aust et al., 2020; Lu et al., 2025; Pham et al., 2023). When employees perceive high levels of CGHRM, the organization’s emphasis on responsibility and common good makes the normative goal frame more salient in interpreting employee stock ownership, thereby strengthening felt responsibility (Pearce and Gregersen, 1991) and reducing the likelihood of engaging in unethical pro-organizational behavior. Conversely, when employees perceive low levels of CGHRM, they are more likely to interpret employee stock ownership from the perspective of personal gains and performance outcomes, making the gain goal frame more salient and strengthening bottom-line mentality (Greenbaum et al., 2012, 2023), which in turn increases the likelihood of engaging in unethical pro-organizational behavior. Thus, goal-framing theory explains how CGHRM shapes employees’ interpretations of employee stock ownership and influences the process through which employee stock ownership affects unethical pro-organizational behavior via felt responsibility and bottom-line mentality.

### Employee stock ownership level, perceived CGHRM practices and felt responsibility

Felt responsibility refers to an internalized psychological state in which employees perceive that they are personally responsible for the outcomes of their work and the effective performance of their work roles (Pearce and Gregersen, 1991; Fuller et al., 2006; Arain et al., 2019). This sense of responsibility does not arise merely from employees’ possession of formal rights or organizational positions; rather, it depends on whether employees perceive that they assume a meaningful organizational role and that their actions can influence organizational functioning and outcomes (Fuller et al., 2006; Arain et al., 2019). Increases in employee stock ownership provide employees with greater ownership interests, enabling them to share more extensively in organizational value creation and operating outcomes and making them direct stakeholders in organizational results (Pierce et al., 1991; Pendleton and Robinson, 2010). Drawing on goal-framing theory (Lindenberg and Foss, 2011), employee stock ownership contains both economic benefit and responsibility-related attributes, which may activate different goal frames. Specifically, the normative goal frame directs employees’ attention toward whether their behaviors conform to the responsibilities associated with organizational membership and encourages behavioral judgments based on responsibility and appropriateness (Lindenberg and Foss, 2011). Therefore, higher levels of employee stock ownership strengthen employees’ connection with organizational outcomes, providing an important foundation for the development of felt responsibility.

CGHRM refers to an HRM orientation that goes beyond a narrow focus on economic performance by incorporating employee well-being, stakeholder interests, and broader social value into HRM practices (Aust et al., 2020; Lu et al., 2025; Pham et al., 2023). Drawing on goal-framing theory (Lindenberg and Foss, 2011), CGHRM influences the relative salience of different goal frames when employees respond to different levels of employee stock ownership. When employees perceive high levels of CGHRM, the organization’s emphasis on common good, long-term development, and responsibility strengthens the normative goal frame, making employees more attentive to the responsibility implications associated with higher levels of employee stock ownership. Under such conditions, employees are more likely to view themselves as important participants in organizational functioning and perceive that they should personally take responsibility for organizational outcomes, thereby strengthening the positive relationship between employee stock ownership level and felt responsibility. Conversely, when employees perceive low levels of CGHRM, the organization places less emphasis on responsibility and common good considerations, weakening the influence of the normative goal frame. Therefore, the positive relationship between employee stock ownership level and felt responsibility is attenuated. Accordingly, we propose:

Hypothesis 1: Perceived CGHRM practices moderate the positive relationship between employee stock ownership level and felt responsibility, such that this relationship is stronger when perceived CGHRM practices are high rather than low.

### Employee stock ownership level, perceived CGHRM practices and bottom-line mentality

Bottom-line mentality refers to a psychological state in which employees focus exclusively on organizational bottom-line outcomes while disregarding competing work goals and broader organizational concerns (Greenbaum et al., 2012, 2023). Bottom-line mentality is not entirely a stable individual tendency but can also emerge as a situationally influenced psychological state, such that employees are more likely to direct their attention toward economic performance outcomes when these outcomes become more salient in a given context (Greenbaum et al., 2023; Mawritz et al., 2024b). Higher levels of employee stock ownership strengthen the connection between employees’ personal economic returns and organizational performance, making economic outcomes more salient when employees evaluate organizational activities (Pierce et al., 1991; McConville et al., 2016). Drawing on goal-framing theory (Lindenberg and Foss, 2011), higher levels of employee stock ownership may increase the salience of the gain goal frame, thereby directing employees’ attention toward the personal economic outcomes associated with organizational performance. Therefore, higher levels of employee stock ownership may increase employees’ attention to economic outcomes related to organizational performance and provide a psychological basis for the development of bottom-line mentality.

Based on goal-framing theory (Lindenberg and Foss, 2011), the extent to which employee stock ownership level translates into bottom-line mentality depends on the organizational context in which it is embedded. When employees perceive high levels of CGHRM, the organization’s emphasis on employee well-being, stakeholder interests, and long-term development encourages employees to pay greater attention to organizational goals beyond economic performance (Aust et al., 2020; Lu et al., 2025; Pham et al., 2023). Under such conditions, although employee stock ownership still strengthens the connection between personal economic interests and organizational performance, this economic connection is no longer the sole basis for employees’ evaluations of their ownership role. The multi-goal environment shaped by CGHRM reduces the dominance of the gain goal frame, making employees more likely to consider other organizational goals while pursuing organizational performance. Therefore, the attention to economic outcomes resulting from higher levels of employee stock ownership is less likely to further develop into an exclusive focus on bottom-line outcomes, thereby weakening the positive relationship between employee stock ownership level and bottom-line mentality.

By contrast, when employees perceive low levels of CGHRM, the organizational context places less emphasis on responsibilities beyond economic performance and broader organizational goals, making the economic connection brought by employee stock ownership more likely to become the primary focus. Under such conditions, the gain goal frame is more likely to become dominant, leading employees to prioritize improving organizational performance and devote less attention to other organizational goals (Mawritz, Greenbaum, et al., 2024). Because bottom-line mentality involves not only an emphasis on economic performance but, more importantly, the neglect of other important goals and organizational concerns in pursuit of bottom-line outcomes (Greenbaum et al., 2012, 2023), lower levels of CGHRM strengthen the positive relationship between employee stock ownership level and bottom-line mentality. Taken together, CGHRM constrains the extent to which employee stock ownership level translates into bottom-line mentality. Therefore, we propose:

Hypothesis 2: Perceived CGHRM practices moderate the positive relationship between employee stock ownership level and bottom-line mentality, such that this relationship is stronger when perceived CGHRM practices are low rather than high.

### Felt responsibility and unethical pro-organizational behavior

Unethical pro-organizational behavior refers to actions undertaken by employees to benefit their organization or its members that violate core societal values, laws, or moral standards (Umphress et al., 2010; Umphress and Bingham, 2011). Prior research suggests that whether employees engage in unethical pro-organizational behavior depends not only on their motivation to help the organization but also on how they understand organizational interests and evaluate the appropriateness of the means used to pursue those interests (Umphress and Bingham, 2011; Chen et al., 2016). Drawing on goal-framing theory (Lindenberg and Foss, 2011), when the normative goal frame becomes dominant, employees are more likely to focus on whether their behaviors are appropriate and consistent with their obligations as organizational members rather than merely considering the outcomes generated by their actions (Lindenberg and Foss, 2011). Therefore, we propose that felt responsibility, as an internalized sense of personal accountability for work-related outcomes (Fuller et al., 2006), encourages employees to evaluate not only the outcomes of their actions but also whether the means used are consistent with appropriate standards and responsibilities, thereby reducing unethical pro-organizational behavior.

Specifically, first, employees with stronger felt responsibility perceive themselves as personally accountable for the outcomes of their work and the consequences of their actions (Pearce and Gregersen, 1991; Fuller et al., 2006). Therefore, they consider not only whether a particular action can generate immediate benefits but also whether the means used to achieve these outcomes are consistent with appropriate standards and responsibilities. Although unethical pro-organizational behavior may produce short-term benefits for the organization, its violation of ethical norms may create potential harm to organizational reputation, legitimacy, and stakeholder relationships (Umphress et al., 2010; Umphress and Bingham, 2011). Employees with stronger felt responsibility are therefore more likely to recognize that short-term organizational gains obtained through unethical means do not necessarily reflect responsible actions, making them less likely to engage in unethical pro-organizational behavior. Second, unethical pro-organizational behavior often relies on moral disengagement, whereby employees emphasize the pro-organizational purpose of their actions, minimize the harm caused, or displace personal responsibility to justify unethical means (Umphress and Bingham, 2011; Chen et al., 2016). When employees experience stronger felt responsibility, they are more likely to view the consequences of their actions as personally accountable outcomes and maintain stricter standards regarding how organizational goals should be achieved (Pearce and Gregersen, 1991; Fuller et al., 2006). Therefore, they are less able to justify unethical means by emphasizing pro-organizational intentions, minimizing potential harm, or shifting responsibility, thereby reducing their likelihood of engaging in unethical pro-organizational behavior. Based on the preceding reasoning, we propose the following hypothesis:

Hypothesis 3: Felt responsibility is negatively related to unethical pro-organizational behavior.

### Bottom-line mentality and unethical pro-organizational behavior

Drawing on goal-framing theory (Lindenberg and Foss, 2011), when the gain goal frame becomes dominant, employees are more likely to focus on resource acquisition, performance outcomes, and gain-related goals, while paying less attention to whether their behaviors are consistent with broader responsibilities and normative requirements (Lindenberg and Foss, 2011). Bottom-line mentality reflects employees’ exclusive focus on organizational bottom-line outcomes and their neglect of competing goals and broader organizational concerns (Greenbaum et al., 2012, 2023), thereby representing the dominance of a gain-oriented focus in behavioral judgments. Accordingly, we further propose that employees’ bottom-line mentality promotes unethical pro-organizational behavior.

Specifically, first, bottom-line mentality narrows employees’ attention toward bottom-line outcomes while reducing the salience of ethical considerations and stakeholder concerns in their decision making. Employees with stronger bottom-line mentality tend to regard improving organizational performance as the primary objective and devote comparatively less attention to competing demands (Greenbaum et al., 2012, 2023; Mawritz et al., 2024a). When considering whether to engage in unethical pro-organizational behavior, they are therefore more likely to focus on the performance benefits that such behavior may generate for the organization while paying less attention to its ethical implications and potential harm to stakeholders. This attentional imbalance increases their likelihood of engaging in such behavior (Umphress et al., 2010; Umphress and Bingham, 2011).

Second, bottom-line mentality facilitates the moral justification of unethical means. Because employees with stronger bottom-line mentality evaluate actions primarily based on their contribution to organizational performance, they are more likely to perceive performance gains as outweighing the ethical impropriety of the means used to achieve them (Greenbaum et al., 2012; Zhang et al., 2020). They may consequently engage in moral disengagement by emphasizing the pro-organizational purpose of their behavior, minimizing potential harm, or reducing their sense of personal responsibility, thereby alleviating moral concerns and self-condemnation associated with unethical conduct (Chen et al., 2016; Zhang et al., 2020). Consistent with this reasoning, prior research has shown that an excessive focus on bottom-line outcomes not only increases employees’ general unethical behavior but also encourages them to engage in unethical pro-organizational behavior to achieve organizational performance goals (Babalola et al., 2021; Zhang et al., 2020; Farasat and Azam, 2022; Mawritz et al., 2024a). Based on the preceding reasoning, we propose the following hypothesis:

Hypothesis 4: Bottom-line mentality is positively related to unethical pro-organizational behavior.

### An integrative model

Drawing on goal-framing theory, employees’ responses to organizational arrangements depend on which goal frame becomes more salient in a given context (Lindenberg, 2001; Lindenberg and Steg, 2007). Integrating Hypotheses 1 and 3 as well as Hypotheses 2 and 4, we further propose a CGHRM-contingent dual-path conditional indirect-effect model. Specifically, when employees perceive high levels of CGHRM, the organization’s emphasis on employee well-being, stakeholder interests, and long-term development makes the normative goal frame more salient (Aust et al., 2020; Lu et al., 2025; Pham et al., 2023). Under such conditions, employees are more likely to attend to the responsibility-related meaning of employee stock ownership and perceive their ownership role as involving greater personal accountability for work outcomes, thereby strengthening felt responsibility (Pierce et al., 1991; Fuller et al., 2006). Felt responsibility subsequently encourages employees to evaluate not only the outcomes of their actions but also whether the means used to achieve these outcomes are consistent with appropriate standards and responsibilities, reducing their likelihood of engaging in unethical pro-organizational behavior (Pearce and Gregersen, 1991; Fuller et al., 2006; Umphress and Bingham, 2011).

By contrast, when employees perceive low levels of CGHRM, the organizational context places less emphasis on responsibilities beyond economic performance and broader organizational objectives, making the gain goal frame more salient (Lindenberg and Foss, 2011). Under such conditions, employees are more likely to focus on the economic outcomes associated with employee stock ownership and use organizational performance gains as an important criterion for evaluating their actions, thereby strengthening bottom-line mentality (Greenbaum et al., 2012, 2023; Mawritz et al., 2024a). Bottom-line mentality subsequently directs employees’ attention toward organizational performance benefits while reducing attention to ethical standards and stakeholder concerns, making employees more likely to justify unethical means based on pro-organizational intentions and ultimately increasing unethical pro-organizational behavior (Umphress et al., 2010; Umphress and Bingham, 2011; Chen et al., 2016). Therefore, CGHRM shapes the extent to which employee stock ownership relates to unethical pro-organizational behavior through distinct psychological pathways.

Hypothesis 5: Perceived CGHRM practices moderate the indirect effect of employee stock ownership level on unethical pro-organizational behavior through felt responsibility, such that the negative indirect effect is stronger when perceived CGHRM practices are high rather than low.

Hypothesis 6: Perceived CGHRM practices moderate the indirect effect of employee stock ownership level on unethical pro-organizational behavior through bottom-line mentality, such that the positive indirect effect is stronger when perceived CGHRM practices are low rather than high.

## Methods

### Sample and procedure

Our data were collected from employees working in three enterprises located in Beijing, China. To protect organizational confidentiality, the identities of the participating firms are not disclosed. China provides an appropriate empirical context for the present study because sustainable development has become increasingly embedded in the country’s institutional and organizational agendas, encouraging firms to incorporate broader social, environmental, and stakeholder concerns into their management and HRM practices (Lu et al., 2023; Lu et al., 2025). Related research in emerging economies likewise suggests that national sustainable development agendas can encourage organizations to integrate common-good considerations into their HRM systems (Lu et al., 2023; Lu et al., 2025; Pham et al., 2023).

The three participating enterprises were particularly suitable for testing our model because they had formally implemented employee stock ownership arrangements through employee holding platforms, under which employees held company shares according to organizationally established criteria rather than individual investment decisions. Specifically, employees obtained company shares through formal organizational mechanisms, such as promotion and performance-based rewards, rather than purchasing shares voluntarily. The employee stock ownership arrangements adopted by these enterprises were implemented through holding platforms, with a three-year vesting period. During the employment period, employees were subject to holding restrictions and were not permitted to transfer their shares to external parties privately. When employees left the organizations, the companies repurchased their shares according to the established arrangements. Moreover, employees could not freely trade their shares in the market, and their ownership interests were primarily realized through organizationally determined arrangements rather than immediate liquidity. Therefore, variation in the value of shares held by employees primarily reflected differences in their ownership levels within the organizationally established employee stock ownership system. The value of shares held by individual employees varied substantially, allowing us to examine differences in employees’ stock ownership levels within these organizations. At the same time, these enterprises had implemented HRM practices consistent with key elements of CGHRM, including fair employment relationships, employee participation and workplace democracy, employee safety and well-being, meaningful work, fair compensation, and contributions to society. The coexistence of employee stock ownership arrangements and common-good-oriented HRM practices provided an appropriate setting for examining how perceived CGHRM practices shape employees’ interpretations of employee stock ownership level and, consequently, its psychological and behavioral consequences.

Prior to data collection, we contacted the HR managers of the participating enterprises and explained the purpose and procedures of the study. With assistance from the HR departments, full-time employees who held company shares through the formal employee stock ownership arrangements and their coworkers were invited to participate voluntarily. At the outset, 360 employee–coworker dyads participated in the study, with each focal employee paired one-to-one with a coworker. The HR departments assisted only in identifying and contacting eligible participants and did not have access to individual survey responses.

This study was reviewed and approved by the Ethics Committee of the Business Administration Department, School of Economics and Management, Beijing Jiaotong University (Approval No. 20260506). All participants provided informed consent prior to data collection. Focal employees were informed in advance that their matched coworkers would identify them by name when providing behavioral ratings at Wave 3 and voluntarily consented to this procedure. To protect respondent confidentiality while enabling data matching, each participant was assigned a unique identification code before data collection. These codes were used solely to match employee responses across survey waves and link them with the corresponding coworker ratings and contained no names, employee numbers, telephone numbers, or other personally identifiable information. Participants were also assured that individual responses would not be disclosed to supervisors, coworkers, HR personnel, or other members of their organizations. All survey data were treated confidentially, and research findings were reported only in aggregate form.

Data were collected in three waves, with approximately 1 month between consecutive waves. This temporal separation, combined with the use of multiple data sources, was intended to establish a clearer temporal ordering among the focal variables and reduce concerns about common method variance (Podsakoff et al., 2012). At Wave 1, employees reported their employee stock ownership levels, perceived CGHRM practices, and demographic and work-related control variables. Approximately 1 month later, at Wave 2, the same employees reported their felt responsibility and bottom-line mentality. At Wave 3, conducted approximately 1 month after Wave 2, the matched coworkers rated the focal employees’ unethical pro-organizational behavior. Coworker ratings were used because coworkers typically interact frequently and continuously with focal employees in their daily work and therefore have greater opportunities to observe their actual workplace behaviors, including behaviors that may not be readily visible to supervisors (Fox et al., 2007). In addition, because unethical workplace behavior is a sensitive construct, coworker ratings reduce reliance on employee self-reports and provide an independent source of behavioral assessment (Berry et al., 2012). Unique identification codes were used throughout the three waves to match employee responses over time and link them with the corresponding coworker ratings.

After the three waves of data collection, we matched and screened the responses. Questionnaires that could not be successfully matched across waves, contained substantial missing information, or exhibited evident careless or patterned responding were excluded. This process resulted in a final sample of 303 matched employee–coworker dyads, representing 84.2% of the initial matched sample.

Among the focal employees, 53.1% were female and 46.9% were male. In terms of age, 4.6% were 21–30 years old, 19.1% were 31–40, 35.3% were 41–50, 23.8% were 51–60, and 17.2% were over 60. With respect to education, 3.3% had a high school education or below, 14.9% held an associate degree, 48.2% held a bachelor’s degree, 21.8% held a master’s degree, and 11.9% held a doctoral degree. Regarding organizational tenure, 16.2% had worked for their current organization for less than 1 year, 17.5% for 1–3 years, 40.9% for 3–5 years, 15.5% for 5–10 years, and 9.9% for more than 10 years. In terms of functional area, 8.6% worked in human resources, 10.9% in finance, 24.1% in marketing or sales, 17.8% in production or operations, 14.9% in research and development or technical functions, 13.9% in administration or general management, 5.3% in senior management, and 4.6% in other functions. Finally, with respect to coworker interaction frequency, 13.2% interacted with their matched coworkers once a week or less, 15.2% two or three times per week, 16.5% almost every day, 31.7% once or twice per day, and 23.4% several times per day.

### Measures

The multi-item measures were adapted from previously validated scales. Because the original scales were developed in English, we followed Brislin’s (1980) translation and back-translation procedure to ensure linguistic and conceptual equivalence. One bilingual researcher translated the original items into Chinese, and another bilingual researcher independently back-translated them into English. The research team then compared the back-translated items with the original versions and resolved any discrepancies through discussion. Unless otherwise indicated, all constructs were measured using seven-point Likert-type scales ranging from 1 (“strongly disagree”) to 7 (“strongly agree”), with higher scores indicating higher levels of the corresponding constructs.

#### Employee stock ownership level

Following French and Rosenstein (1984), employee stock ownership level was measured using a single item asking employees to report the approximate monetary value of the company shares they currently held. Respondents reported the value in units of RMB 1,000. This measure captures employees’ economic stake in the organization by reflecting the financial value of their equity holdings. In the final sample, the average value of employee stock ownership level was 30.04 thousand RMB (SD = 15.20), ranging from 10.00 to 65.70 thousand RMB. The distribution of employee stock ownership level did not show substantial deviations from normality, with a skewness value of 0.47 and a kurtosis value of −0.89 (Kline, 2016).

#### Perceived CGHRM practices

Perceived CGHRM practices were measured using the four-item scale developed by Pham et al. (2023). The scale captures employees’ perceptions of the extent to which their organizations’ HR practices reflect common-good-oriented principles, including fair employment, employee participation and workplace democracy, employee safety and meaningful work, and fair compensation and social well-being. A sample item was: “I believe that my company provides employees at all levels with equal and fair employment relationships.” Cronbach’s alpha was 0.929.

#### Felt responsibility

Felt responsibility was measured using four items adapted from Pearce and Gregersen (1991). The original reverse-coded fourth item was reworded positively to ensure consistent item direction. A sample item was “I feel a very high degree of personal responsibility for the work I do on this job.” Cronbach’s alpha was 0.921.

#### Bottom-line mentality

Employee bottom-line mentality was measured using the four-item scale developed by Greenbaum et al. (2012). A sample item was: “I treat the bottom line as more important than anything else.” Cronbach’s alpha was 0.934.

#### Unethical pro-organizational behavior

Coworkers rated the focal employees’ unethical pro-organizational behavior using an adapted six-item scale developed by Umphress et al. (2010). A sample item was: “If it would help the organization, [Employee’s name] would misrepresent the truth to make the organization look good.” Cronbach’s alpha was 0.935.

#### Control variables

We controlled for employees’ demographic and work-related characteristics, including gender, age, education, and organizational tenure. In addition, we controlled for functional department and coworker interaction frequency to account for potential confounding effects in the analyses. Following Fehr et al. (2025), coworker interaction frequency was assessed with a single item asking respondents how frequently they interacted with their coworkers, ranging from 1 (“once a week or less”) to 5 (“several times a day”). Functional department was treated as a categorical variable and represented using dummy variables.

## Results

### Confirmatory factor analyses

**W**e conducted confirmatory factor analysis (CFA) to evaluate the discriminant validity of the multi-item constructs. Employee stock ownership level was not included in the CFA because it was measured using a single item reflecting the monetary value of employees’ actual shareholdings and was therefore treated as an observed variable rather than a latent construct represented by multiple indicators (Brown, 2015; Kline, 2016). As presented in Table 1, the hypothesized four-factor model comprising perceived CGHRM practices, bottom-line mentality, felt responsibility, and unethical pro-organizational behavior exhibited a satisfactory fit to the data (χ^2^ = 262.490, df = 129, χ^2^/df = 2.035, CFI = 0.971, TLI = 0.965, RMSEA = 0.058, SRMR = 0.028). Compared with the alternative factor structures, the hypothesized four-factor model provided the best representation of the measurement structure. These findings support the discriminant validity of the four multi-item constructs.

**Table 1:** Confirmatory factor analysis.

Model	χ^2^	df	χ^2^/df	CFI	TLI	RMSEA	SRMR
Four-factor model: PCGHRMP, BLM, FR, UPB	262.490	129	2.035	0.971	0.965	0.058	0.028
Three-factor model: PCGHRMP, FR + BLM, UPB	1298.392	132	9.836	0.743	0.702	0.171	0.148
Two-factor model: PCGHRMP + FR + BLM, UPB	2122.710	134	15.841	0.561	0.499	0.221	0.202
One-factor model: PCGHRMP+BLM + FR + UPB	3015.005	135	22.333	0.364	0.280	0.265	0.230

*N* = 303. PCGHRMP = perceived common-good human resource management practices; BLM = bottom-line mentality; FR = felt responsibility; UPB = unethical pro-organizational behavior.

### Unmeasured latent method factor technique

Given that perceived CGHRM practices, felt responsibility, and bottom-line mentality were self-reported by focal employees, we further assessed common method variance using an unmeasured latent method factor approach. Specifically, we added an orthogonal method factor to the original four-factor measurement model and allowed all items of the three employee-reported constructs to load on this factor. The method-factor model showed improved fit (χ^2^ = 206.349, df = 117, CFI = 0.980, TLI = 0.974, RMSEA = 0.050, SRMR = 0.024). However, the variance attributable to the method factor was limited, and the standardized loadings of the substantive factors remained largely stable after the method factor was introduced. Thus, although CMV cannot be completely ruled out, it is unlikely to have materially affected the measurement results.

### Common method variance

**T**o further assess the potential influence of common method variance (CMV), we conducted Harman’s single-factor test (Podsakoff and Organ, 1986). All multi-item measures were entered simultaneously into an unrotated exploratory factor analysis. The results indicated that the first factor accounted for 33.051% of the total variance, below the commonly used 40% criterion. Together with the temporal separation, multi-source design, and results from the unmeasured latent method factor technique, these findings suggest that common method variance is unlikely to fully account for the observed relationships.

### Firm-level clustering

Because employees were nested within three firms, we calculated ICC(1) and ICC(2) for the focal variables to assess the extent of firm-level clustering (Bliese, 2000; LeBreton and Senter, 2008). The ICC(1) values were 0.038 for employee stock ownership level, 0.019 for felt responsibility, and 0.038 for bottom-line mentality, with corresponding ICC(2) values of 0.797, 0.664, and 0.798, respectively. The ICC(1) estimates for perceived CGHRM practices and unethical pro-organizational behavior were approximately zero, indicating negligible between-firm variance. Because the focal constructs were conceptualized and measured at the individual level and the sample included only three firms, we retained the individual level of analysis rather than aggregating these variables to the firm level. Nevertheless, we further addressed potential firm-level clustering concerns by introducing firm dummy variables as fixed effects in robustness analyses.

### Discriminant validity

Discriminant validity was further assessed using the heterotrait–monotrait ratio (HTMT). Following Henseler et al. (2015), we adopted the conservative threshold of 0.85 because the focal constructs are theoretically distinct. All HTMT values were well below this threshold, ranging from 0.102 to 0.439. In particular, the HTMT value between felt responsibility and bottom-line mentality was 0.116, providing further evidence of discriminant validity.

### Multicollinearity diagnostics

We further examined multicollinearity across the regression equations used to test Hypotheses 1–6. Consistent with conventional VIF diagnostics, and recognizing that fixed cutoffs should be interpreted cautiously, all VIF values were below the commonly cited threshold of 10 (O’Brien, 2007). Across all predictors, VIF values ranged from 1.04 to 4.86, while those for the focal theoretical variables ranged from 1.19 to 1.69. Importantly, the VIF values for felt responsibility and bottom-line mentality were only 1.52 and 1.30. These results suggest that multicollinearity was unlikely to pose a serious concern.

### Descriptive statistics

Table 2 reports the descriptive characteristics of the variables under investigation, including their means, standard deviations, and correlation coefficients. Overall, the observed associations among the focal constructs were in accordance with the expected theoretical relationships, suggesting that the variables were related in the anticipated directions.

**Table 2:** Descriptive statistics, correlations, and Cronbach’s α reliability.

Variable	Means	SD	1	2	3	4	5	6	7	8	9	10
1. Gender	1.53	0.500	-									
2. Age	4.30	1.103	0.103	*-*								
3. Education	3.24	0.958	−0.061	0.133*	*-*							
4. Tenure	2.85	1.165	0.053	−0.046	0.094	*-*						
5. Interaction Frequency	3.37	1.343	−0.003	0.024	−0.082	−0.040	–					
6. ESO	30.039	15.202	0.049	0.059	0.070	−0.041	0.018	–				
7. BLM	3.558	1.920	0.071	0.033	0.026	0.133*	−0.108	0.411**	***(0.934)***			
8. FR	2.926	1.345	0.102	0.080	−0.010	−0.229**	−0.029	0.509**	0.107	***(0.921)***		
9. UPB	3.851	1.592	−0.029	0.059	0.091	0.312**	−0.025	−0.341**	0.094	−0.407**	***(0.935)***	
10. PCGHRMP	3.266	1.383	0.056	0.002	−0.013	−0.094	−0.145*	0.296**	0.166**	0.315**	−0.125*	***(0.929)***

*N* = 303. Internal consistent reliability (alpha) coefficients are shown along the diagonal in bold italics. Gender, 1 = male, and 2 = female. Age, 1 = 20 years old or younger, 2 = 21–30 years old, 3 = 31–40 years old, 4 = 41–50 years old, 5 = 51–60 years old, 6 = Over 60 years. Education, 1 = High school education or below, 2 = Associate degree, 3 = Bachelor’s degree, 4 = Master’s degree, 5 = Doctoral degree. Tenure, 1 = Less than 1 year, 2 = 1–3 years, 3 = 3–5 years, 4 = 5–10 years, 5 = More than 10 years. Functional department was measured as an eight-category nominal variable and was dummy coded in all regression analyses; therefore, it was not included as a single variable in the correlation matrix. **p < 0.01, *p < 0.05, same for following table. ESO = employee stock ownership level; BLM = bottom-line mentality; FR = felt responsibility; UPB = unethical pro-organizational behavior; PCGHRMP = perceived common-good human resource management practices.

### Hypothesis testing

To test the proposed hypotheses, we estimated the moderated mediation model using Mplus 8.3 (Muthén and Muthén, 1998–2017) with maximum likelihood estimation. Employee stock ownership and perceived CGHRM practices were grand-mean centered before constructing their interaction term. We used 5,000 bootstrap resamples to obtain bias-corrected 95% confidence intervals for the conditional indirect effects and the differences between these effects at high and low levels of perceived CGHRM practices. The results are presented in Tables 3, 4.

**Table 3:** Results of path analyses.

Variable	Felt responsibility			Bottom-line mentality			Unethical pro-organizational behavior		
	Estimate	SE	95% CI	Estimate	SE	95% CI	Estimate	SE	95% CI
Gender	0.190	0.126	[−0.056, 0.437]	0.091	0.200	[−0.301, 0.482]	−0.056	0.158	[−0.365, 0.254]
Age	0.053	0.057	[−0.058, 0.164]	0.040	0.090	[−0.136, 0.216]	0.135	0.071	[−0.005, 0.274]
Education	−0.018	0.065	[−0.146, 0.110]	−0.067	0.103	[−0.270, 0.136]	0.111	0.082	[−0.049, 0.271]
Tenure	−0.218***	0.053	[−0.356, −0.113]	0.235**	0.085	[0.087, 0.420]	0.295***	0.069	[0.158, 0.431]
HR	0.414	0.353	[−0.277, 1.106]	−0.389	0.560	[−1.487, 0.709]	0.231	0.444	[−0.639, 1.101]
FIN	0.206	0.342	[−0.464, 0.877]	−0.490	0.543	[−1.555, 0.574]	0.541	0.426	[−0.294, 1.375]
MKT	−0.019	0.311	[−0.629, 0.591]	−0.624	0.494	[−1.592, 0.344]	0.463	0.389	[−0.298, 1.225]
PROD	0.046	0.320	[−0.582, 0.673]	−0.580	0.508	[−1.576, 0.416]	0.242	0.401	[−0.545, 1.029]
R&D	0.117	0.326	[−0.521, 0.756]	−0.241	0.517	[−1.254, 0.773]	0.309	0.406	[−0.487, 1.105]
ADM	−0.233	0.327	[−0.875, 0.408]	−0.785	0.520	[−1.803, 0.234]	0.676	0.412	[−0.132, 1.484]
SM	−0.281	0.386	[−1.037, 0.476]	−0.156	0.613	[−1.357, 1.045]	0.373	0.485	[−0.577, 1.323]
Interaction frequency	−0.054	0.047	[−0.146, 0.038]	−0.110	0.074	[−0.256, 0.036]	0.018	0.058	[−0.131, 0.131]
ESO	0.036***	0.004	[0.027, 0.044]	0.056***	0.007	[0.043, 0.070]	−0.034***	0.006	[−0.047, −0.022]
PCGHRMP	0.085	0.050	[−0.014, 0.184]	0.126	0.080	[−0.031, 0.283]			
ESO × PCGHRMP	0.011***	0.003	[0.006, 0.016]	−0.013**	0.004	[−0.022, −0.005]			
FR							−0.248***	0.070	[−0.384, −0.111]
BM							0.190***	0.045	[0.101, 0.278]
R2	0.387***	0.044		0.242***	0.043		0.311***	0.044	
Adjusted R2	0.355			0.202			0.287		
F	12.08***			6.11***			8.64***		
df	(15, 287)			(15, 287)			(15, 287)		

*N* = 303. Functional department was dummy coded. HR = Human resources; FIN = Finance; MKT = Marketing/Sales; PROD = Production/Operations; R&D = Research and Development/Technical functions; ADM = Administration/General management; SM = Senior management; OTH = Other functions (reference group); ESO = employee stock ownership level; PCGHRMP = perceived CGHRM practices; FR = felt responsibility; BM = bottom-line mentality. **p < 0.01, ***p < 0.001.

**Table 4:** Summary of direct and indirect effects at different values of perceived CGHRM practices.

Predictor	Effect	SE	95% CI
Conditional direct effect(s)			
M2: Felt responsibility			
Low perceived CGHRM practices	0.021	0.006	[0.008, 0.033]
High perceived CGHRM practices	0.051	0.005	[0.041, 0.061]
Difference	0.030	0.008	[0.014, 0.047]
Conditional direct effect(s)			
M1: Bottom-line mentality			
Low perceived CGHRM practices	0.075	0.010	[0.055, 0.094]
High perceived CGHRM practices	0.038	0.008	[0.022, 0.054]
Difference	−0.037	0.012	[−0.067, −0.013]

Predictor	Effect	BootSE	95% CI
Conditional indirect effect(s)			
M2: Felt responsibility			
Low perceived CGHRM practices	−0.005	0.002	[−0.010, −0.002]
High perceived CGHRM practices	−0.013	0.004	[−0.020, −0.006]
Difference	−0.007	0.002	[−0.013, −0.003]
Conditional indirect effect(s)			
M1: Bottom-line mentality			
Low perceived CGHRM practices	0.014	0.004	[0.007, 0.023]
High perceived CGHRM practices	0.007	0.003	[0.003, 0.014]
Difference	−0.007	0.003	[−0.015, −0.002]
Conditional total effect(s) of ESO on UPB			
Low perceived CGHRM practices	−0.025	0.006	[−0.038, −0.013]
Mean	−0.033	0.006	[−0.044, −0.020]
High perceived CGHRM practices	−0.040	0.006	[−0.052, −0.027]
Difference	−0.014	0.004	[−0.023, −0.008]

*N* = 303. BootSE represents bootstrap standard errors based on 5,000 bootstrap resamples. Confidence intervals are bias-corrected 95% bootstrap confidence intervals. ESO = employee stock ownership level; UPB = unethical pro-organizational behavior.

First, we examined the moderating effect of perceived CGHRM practices on the relationship between employee stock ownership level and felt responsibility. The interaction between employee stock ownership level and perceived CGHRM practices was positive and significant (*b* = 0.011, SE = 0.003, 95% CI = [0.006, 0.016], *p* < 0.001), indicating that the positive relationship between employee stock ownership level and felt responsibility became stronger as perceived CGHRM practices increased. Simple slope analyses further showed that the positive relationship between employee stock ownership level and felt responsibility was stronger when perceived CGHRM practices were high (+1 SD; *b* = 0.051, SE = 0.005, 95% CI = [0.041, 0.061]) than when they were low (−1 SD; *b* = 0.021, SE = 0.006, 95% CI = [0.008, 0.033]). The difference between these two conditional effects was significant (*b* = 0.030, SE = 0.008, 95% CI = [0.014, 0.047]). As illustrated in Figure 2, this pattern was consistent with our prediction. Thus, Hypothesis 1 was supported.

**Figure 2:**
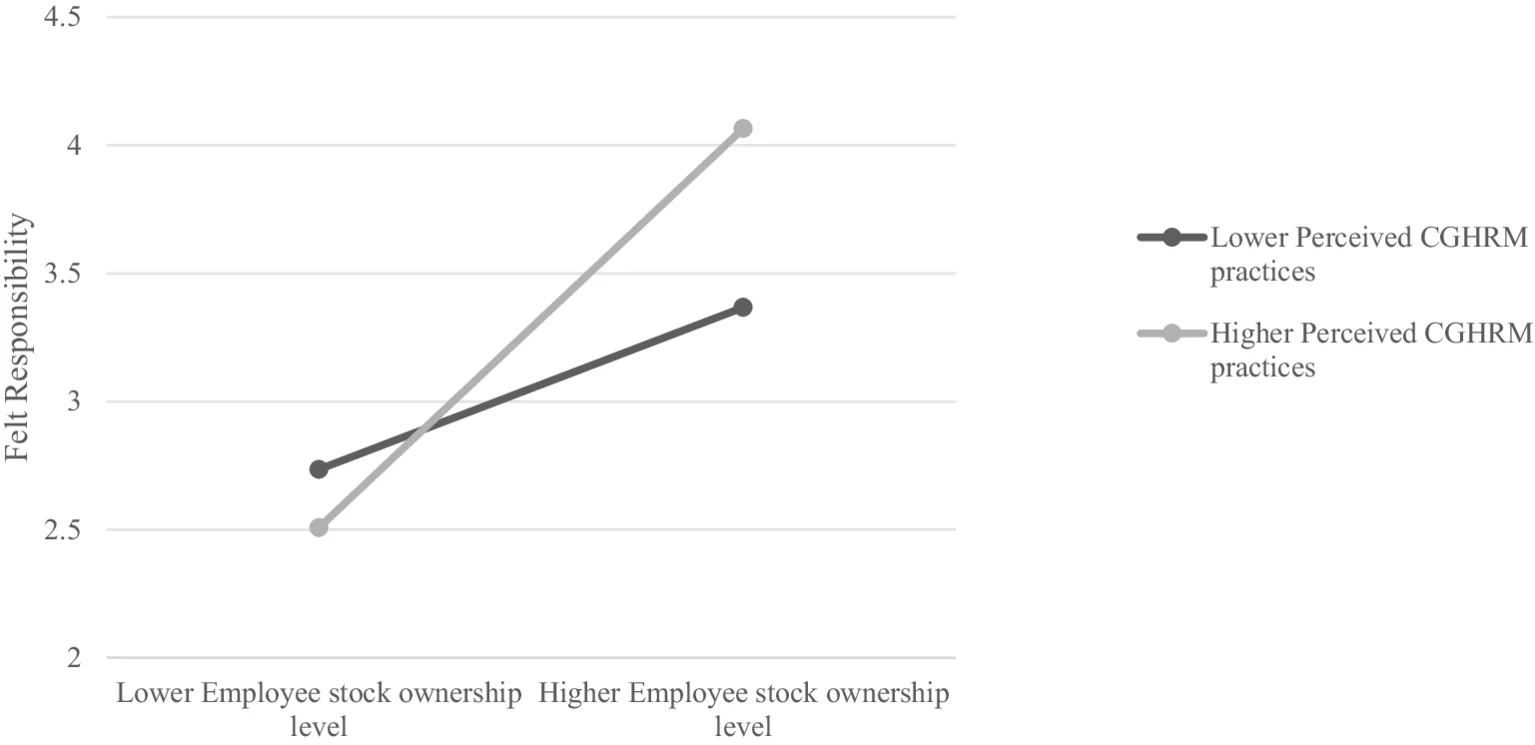
Interaction effect of employee stock ownership level and perceived CGHRM practices on felt responsibility.

In contrast, the interaction between employee stock ownership level and perceived CGHRM practices was negative and significant for bottom-line mentality (*b* = −0.013, SE = 0.004, 95% CI = [−0.022, −0.005], *p* < 0.01), indicating that the positive relationship between employee stock ownership level and bottom-line mentality became weaker as perceived CGHRM practices increased. Simple slope analyses further showed that this positive relationship was stronger when perceived CGHRM practices were low (−1 SD; *b* = 0.075, SE = 0.010, 95% CI = [0.055, 0.094]) than when they were high (+1 SD; *b* = 0.038, SE = 0.008, 95% CI = [0.022, 0.054]). The difference between these two conditional effects was significant (*b* = −0.037, SE = 0.012, 95% CI = [−0.067, −0.013]). As illustrated in Figure 3, this pattern was consistent with our prediction. Thus, Hypothesis 2 was supported.

**Figure 3:**
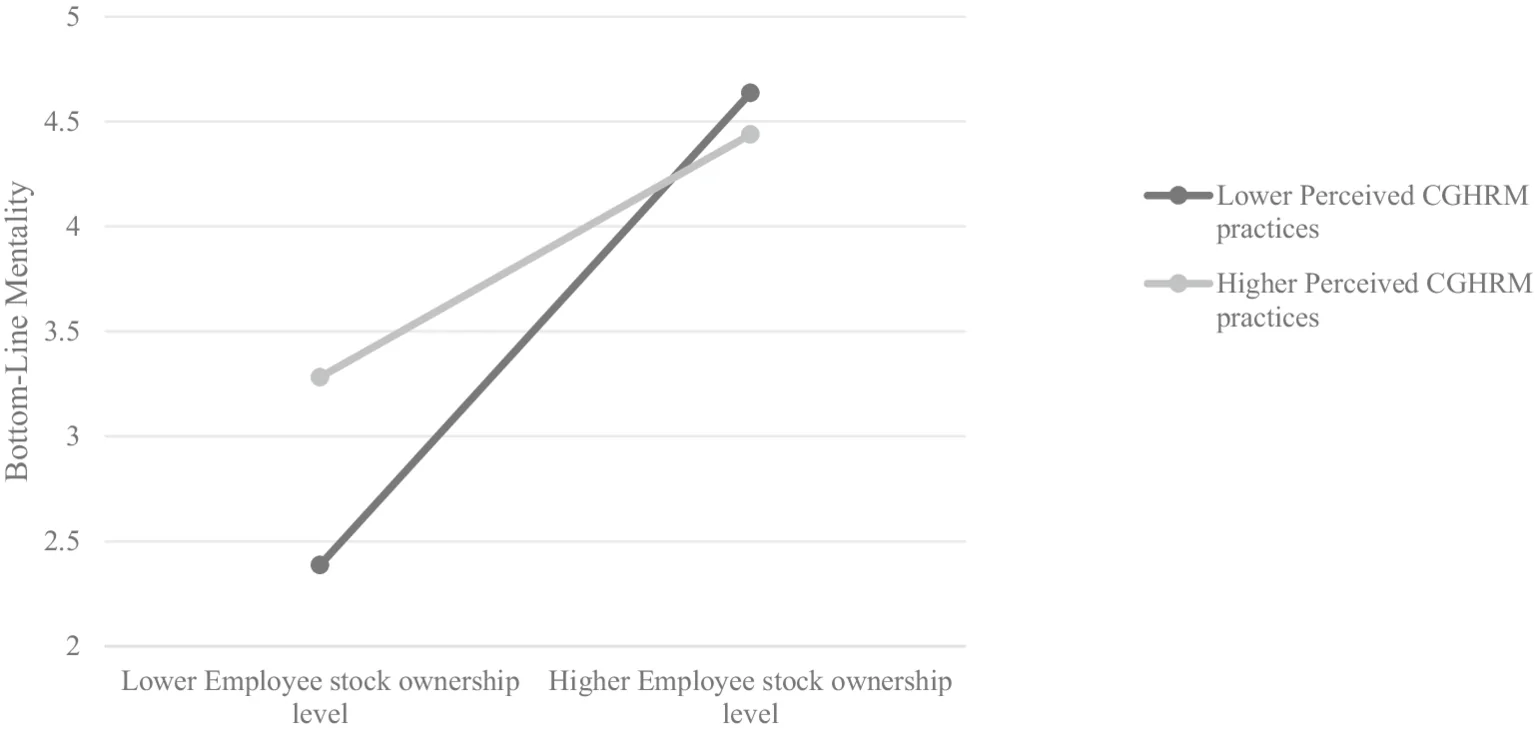
Interaction effect of employee stock ownership level and perceived CGHRM practices on bottom-line mentality.

Second, we examined the relationships between the two psychological mechanisms and unethical pro-organizational behavior. Felt responsibility was negatively associated with unethical pro-organizational behavior (*b* = −0.248, SE = 0.070, 95% CI = [−0.384, −0.111], *p* < 0.001), whereas bottom-line mentality was positively associated with unethical pro-organizational behavior (*b* = 0.190, SE = 0.045, 95% CI = [0.101, 0.278], *p* < 0.001). These results supported Hypotheses 3 and 4.

Finally, we examined the conditional indirect effects of employee stock ownership level on unethical pro-organizational behavior through felt responsibility and bottom-line mentality. Through felt responsibility, the negative indirect effect was stronger when perceived CGHRM practices were high (+1 SD; *b* = −0.013, BootSE = 0.004, 95% CI = [−0.020, −0.006]) than when they were low (−1 SD; *b* = −0.005, BootSE = 0.002, 95% CI = [−0.010, −0.002]). The difference between these conditional indirect effects was significant (*b* = −0.007, BootSE = 0.002, 95% CI = [−0.013, −0.003]), supporting Hypothesis 5. Through bottom-line mentality, the positive indirect effect was stronger when perceived CGHRM practices were low (−1 SD; *b* = 0.014, BootSE = 0.004, 95% CI = [0.007, 0.023]) than when they were high (+1 SD; *b* = 0.007, BootSE = 0.003, 95% CI = [0.003, 0.014]). Consistent with this pattern, the difference between these conditional indirect effects was significant (*b* = −0.007, BootSE = 0.003, 95% CI = [−0.015, −0.002]), supporting Hypothesis 6.

### Robustness checks

First, we examined the sensitivity of our findings to the inclusion of demographic and work-related controls by re-estimating the model with all control variables removed. Omitting these controls did not alter the substantive conclusions. The interaction between employee stock ownership level and perceived CGHRM practices was still positively associated with felt responsibility (*b* = 0.011, SE = 0.003, *p* < 0.001) and negatively associated with bottom-line mentality (*b* = −0.015, SE = 0.005, *p* = 0.003). In turn, felt responsibility remained negatively associated with unethical pro-organizational behavior (*b* = −0.333, SE = 0.071, *p* < 0.001), while bottom-line mentality remained positively associated with unethical pro-organizational behavior (*b* = 0.205, SE = 0.049, *p* < 0.001). The conditional indirect effects also exhibited the same pattern: the high-versus-low difference was significant through felt responsibility (difference = −0.010, BootSE = 0.003, 95% CI [−0.017, −0.005]) and through bottom-line mentality (difference = −0.008, BootSE = 0.004, 95% CI [−0.017, −0.003]). Thus, the hypothesized relationships did not depend on the inclusion of the demographic and work-related controls.

Second, we addressed potential heterogeneity across the three participating firms by introducing two firm dummy variables as fixed effects. After accounting for these firm-level differences, the key relationships were preserved. Employee stock ownership level continued to interact positively with perceived CGHRM practices in predicting felt responsibility (*b* = 0.012, SE = 0.003, *p* < 0.001), whereas the corresponding interaction for bottom-line mentality remained negative (*b* = −0.012, SE = 0.005, *p* = 0.020). Felt responsibility was still negatively associated with unethical pro-organizational behavior (*b* = −0.259, SE = 0.075, *p* = 0.001), while bottom-line mentality retained its positive association with unethical pro-organizational behavior (*b* = 0.187, SE = 0.050, *p* < 0.001). Importantly, the high-versus-low differences in the conditional indirect effects also remained significant for the felt responsibility pathway (difference = −0.008, BootSE = 0.003, 95% CI [−0.015, −0.004]) and the bottom-line mentality pathway (difference = −0.006, BootSE = 0.003, 95% CI [−0.014, −0.001]). These results suggest that the main conclusions are not attributable to systematic differences across firms.

Finally, we assessed whether the results were sensitive to the functional form of the employee stock ownership measure and the potential influence of relatively high ownership values. For this purpose, we replaced the original employee stock ownership level measure with its natural logarithm, ln(ESO + 1), and re-estimated the full model. The alternative specification reproduced the same overall pattern of findings. The interaction between ln(ESO + 1) and perceived CGHRM practices was positive for felt responsibility (*b* = 0.337, SE = 0.093, *p* < 0.001) but negative for bottom-line mentality (*b* = −0.397, SE = 0.153, *p* = 0.009). The associations of felt responsibility (*b* = −0.255, SE = 0.075, *p* = 0.001) and bottom-line mentality (*b* = 0.194, SE = 0.048, *p* < 0.001) with unethical pro-organizational behavior were likewise consistent with the main analyses. Moreover, the difference in the conditional indirect effect between high and low perceived CGHRM practices remained significant through both felt responsibility (difference = −0.237, BootSE = 0.078, 95% CI [−0.428, −0.111]) and bottom-line mentality (difference = −0.213, BootSE = 0.101, 95% CI [−0.469, −0.062]). Taken together, these results indicate that the substantive findings are robust to the logarithmic transformation of employee stock ownership level and are unlikely to be driven by relatively high ownership values. As an additional stringent robustness check, we simultaneously applied the logarithmic transformation of employee stock ownership level and controlled for firm fixed effects. The substantive pattern of results remained unchanged: both interaction effects remained significant, the direct effects of felt responsibility and bottom-line mentality on unethical pro-organizational behavior remained significant and in the expected directions, and the differences between the conditional indirect effects at high versus low levels of perceived CGHRM practices continued to exclude zero.

## Discussion

Although employee stock ownership is widely regarded as an important mechanism for aligning employee and organizational interests, its potential ethical risks remain insufficiently understood. Drawing on multi-wave, multisource field survey data, we examined a conditional dual-path model linking employee stock ownership level to unethical pro-organizational behavior. The results showed that perceived CGHRM practices positively moderated the relationship between employee stock ownership level and felt responsibility, such that this positive relationship was stronger when perceived CGHRM practices were high (H1). In contrast, perceived CGHRM practices negatively moderated the relationship between employee stock ownership level and bottom-line mentality, such that the positive relationship between employee stock ownership level and bottom-line mentality was stronger when perceived CGHRM practices were low (H2).

Further examination of this interaction revealed a noteworthy crossover pattern. When employee stock ownership levels were relatively low, higher perceived CGHRM practices were associated with higher levels of bottom-line mentality; as ownership levels increased, this difference gradually narrowed and eventually reversed. Because this specific pattern was not theoretically predicted *a priori*, we interpret it cautiously. Goal-framing theory suggests that multiple goal frames can be activated simultaneously and that background goals may not only weaken a focal goal but may also enhance its salience when the two are instrumentally related (Lindenberg and Steg, 2007; Lindenberg and Foss, 2011). When employee stock ownership levels are relatively low, the link between employees’ personal economic returns and organizational performance is comparatively weak. Accordingly, ownership-induced gain goals are less likely to constitute the primary basis for employees’ judgments. Under such conditions, higher perceived CGHRM may provide an alternative basis through which bottom-line performance remains relatively salient. CGHRM emphasizes employee well-being, stakeholder interests, long-term development, and the broader common good, the sustained pursuit of which depends in part on the organization’s economic viability and effective functioning (Aust et al., 2020). Thus, when employees’ personal ownership interests are less salient, those perceiving stronger CGHRM may be more likely to view the organization’s economic viability as an important prerequisite for pursuing these broader goals and, consequently, assign greater relative priority to bottom-line performance. By contrast, when both perceived CGHRM and employee stock ownership levels are low, ownership-induced gain goals are relatively weak, while the organizational context places less emphasis on the long-term organizational viability required to sustain common-good goals. Together, these considerations may account for the higher levels of bottom-line mentality observed under higher perceived CGHRM when employee stock ownership levels are relatively low.

As employee stock ownership levels increase, the connection between organizational performance and employees’ personal economic returns becomes increasingly salient, thereby strengthening the gain goals activated by ownership. Under these conditions, the role of perceived CGHRM practices lies primarily in constraining whether such gain goals come to dominate employees’ judgments. When perceived CGHRM practices are low, employee well-being, stakeholder interests, and the common good are less salient as alternative organizational goals. Consequently, as ownership levels rise, the increasingly strong connection between employees’ personal economic interests and organizational performance is more likely to lead employees to prioritize bottom-line performance over competing goals. By contrast, when perceived CGHRM practices are high, multiple organizational goals remain relatively salient, thereby limiting the extent to which gain goals develop into an exclusive focus on bottom-line outcomes. Because bottom-line mentality involves not merely an emphasis on economic performance but, more importantly, the prioritization of bottom-line outcomes over competing goals (Greenbaum et al., 2012, 2023), this mechanism also helps explain why the positive relationship between employee stock ownership level and bottom-line mentality is stronger when perceived CGHRM practices are low.

Accordingly, this crossover pattern suggests that the relationship between perceived CGHRM practices and bottom-line mentality may be more complex than a simple linear main effect. More precisely, our findings do not imply that CGHRM consistently increases or decreases employees’ bottom-line mentality; rather, its role needs to be understood in conjunction with employees’ level of stock ownership. Because this specific pattern was not theoretically predicted *a priori*, we remain cautious in interpreting its theoretical meaning and view it as a supplementary finding that qualifies the moderation captured in H2, rather than as an additional claim regarding the main effect of CGHRM. Future research should examine whether this crossover pattern can be replicated across samples and contexts and identify the psychological processes that may underlie it.

Further, consistent with the proposed dual-path model, felt responsibility was negatively associated with unethical pro-organizational behavior (H3), whereas bottom-line mentality was positively associated with unethical pro-organizational behavior (H4). The negative indirect effect of employee stock ownership level on unethical pro-organizational behavior through felt responsibility was stronger when perceived CGHRM practices were high (H5), whereas the positive indirect effect through bottom-line mentality was stronger when perceived CGHRM practices were low (H6). Taken together, these findings indicate that employee stock ownership level is associated with unethical pro-organizational behavior through psychological pathways operating in opposite directions, and that the relative strength of these pathways varies as a function of perceived CGHRM practices. More importantly, as shown in Table 4, the conditional total effects of employee stock ownership level on unethical pro-organizational behavior were negative at low, mean, and high levels of perceived CGHRM practices. This pattern indicates that, although employee stock ownership level operates through two opposing indirect pathways via felt responsibility and bottom-line mentality, higher ownership levels are, overall, associated with lower levels of unethical pro-organizational behavior. Moreover, as perceived CGHRM practices increase, the felt-responsibility pathway becomes stronger whereas the bottom-line-mentality pathway becomes weaker, resulting in a stronger overall negative association between employee stock ownership level and unethical pro-organizational behavior. Building on these findings, we next discuss the theoretical and practical implications of our study, as well as its limitations and directions for future research.

### Theoretical implications

Our study makes three theoretical contributions. First, we extend the employee stock ownership literature into the domain of employee ethical behavior by revealing the potential ethical consequences associated with differences in ownership levels among employee-owners. Employee stock ownership has long been viewed primarily as an organizational arrangement that aligns employee and organizational interests and thereby improves employee attitudes, behaviors, and organizational performance (Dasilas, 2024; Kim and Han, 2019; Kim et al., 2025; Pendleton and Robinson, 2023). However, this perspective has focused largely on whether employees become more willing to protect organizational interests, while paying considerably less attention to how they pursue those interests. By introducing unethical pro-organizational behavior, our study reveals an overlooked ethical issue in the employee ownership literature: a stronger economic connection between employees and their organizations may strengthen employees’ motivation to protect organizational interests, but such interest alignment does not ensure that the means used to pursue those interests are ethically appropriate. Our findings show that, among employee-owners, higher ownership levels may be associated simultaneously with stronger felt responsibility and stronger bottom-line mentality, which in turn correspond to ethical outcomes in opposite directions. Thus, our study not only extends employee stock ownership research into the domain of employee ethical behavior but also shows that favorable overall consequences of employee–organization interest alignment do not imply that all of its underlying psychological processes carry equally favorable ethical implications.

Second, drawing on goal-framing theory, we identify felt responsibility and bottom-line mentality as two opposing psychological pathways through which employee stock ownership levels are associated with employee ethical behavior. Prior research has largely explained the beneficial consequences of employee ownership through mechanisms such as organizational identification, psychological ownership, and economic incentives (Dasilas, 2024; McConville et al., 2016; O’Boyle et al., 2016; Pierce et al., 1991), but has offered less insight into why the same ownership arrangement may simultaneously generate psychological and ethical consequences in opposite directions. Goal-framing theory provides an integrative perspective for understanding this possibility. Our findings show that higher levels of employee stock ownership are, on the one hand, associated with greater felt responsibility under a normative goal frame, which in turn is associated with lower unethical pro-organizational behavior. On the other hand, higher levels of employee stock ownership are also associated with stronger bottom-line mentality under a gain goal frame, which in turn is associated with higher unethical pro-organizational behavior. By examining these two opposing pathways within a single theoretical framework, our study moves beyond the traditional view of employee ownership as a predominantly beneficial organizational arrangement and demonstrates that the same ownership arrangement may generate divergent ethical consequences. In this way, goal-framing theory provides a more integrative explanation for the potentially double-edged ethical implications of employee stock ownership.

This finding also contributes to recent discussions regarding the heterogeneous motivational foundations of unethical pro-organizational behavior (UPB). Prior research has primarily emphasized the prosocial motives underlying UPB, such as employees’ desire to protect organizational interests; however, recent studies further suggest that self-interested considerations may also motivate employees to engage in behaviors that benefit their organizations while violating ethical standards (Mo et al., 2023; Steele et al., 2024). Our study extends this perspective by demonstrating that employee ownership, as an organizational arrangement, may simultaneously activate responsibility-oriented and economic-interest-oriented psychological processes, thereby explaining the emergence of different motivational foundations underlying UPB.

Third, we identify perceived CGHRM practices as an important boundary condition, further demonstrating that the ethical consequences associated with employee stock ownership depend on the broader HRM context in which ownership is embedded. Goal-framing theory suggests that the relative salience of different goals is not fixed but is shaped by contextual conditions (Lindenberg and Steg, 2007; Lindenberg and Foss, 2011). Consistent with this perspective, our findings show that the pathway linking employee stock ownership level to lower unethical pro-organizational behavior through felt responsibility is stronger when perceived CGHRM practices are high, whereas the pathway linking employee stock ownership level to higher unethical pro-organizational behavior through bottom-line mentality is stronger when perceived CGHRM practices are low. From a goal-framing perspective, this pattern suggests that the emphasis of CGHRM on employee well-being, stakeholder interests, long-term development, and the common good may alter the relative weight of different goal considerations when employees respond to varying levels of ownership, thereby shaping the relative strength of the two psychological pathways. These findings indicate that the ethical consequences associated with employee stock ownership cannot be fully understood independently of the broader HRM system in which ownership arrangements are embedded.

At the same time, this finding also extends the CGHRM literature. Existing research has primarily examined the direct relationships of CGHRM with favorable outcomes such as meaningfulness at work, thriving, ethical behavior, and prosocial behavior (Aust et al., 2020; Lu et al., 2023; Lu et al., 2025; Pham et al., 2023). Our study further suggests that the theoretical role of CGHRM extends beyond its own associations with favorable employee outcomes. CGHRM can also serve as a broader organizational context that conditions the relative strength of the psychological pathways associated with other incentive and HR arrangements. Thus, rather than viewing CGHRM only as a set of HR practices associated with desirable employee outcomes, our findings position it as an important contextual condition for understanding how different organizational arrangements jointly relate to employee behavior.

### Practical implications

Our findings offer three practical implications. First, organizations should consider the ethical implications of employee stock ownership alongside its effects on motivation, retention, productivity, and financial performance. Our findings indicate that higher employee stock ownership levels were overall associated with lower levels of unethical pro-organizational behavior, but this favorable overall association does not imply the absence of potential ethical risks. Accordingly, when evaluating the effectiveness of employee ownership arrangements, organizations should consider not only whether employees achieve desired performance outcomes but also how those outcomes are achieved. More specifically, firms can incorporate behaviors such as concealing compliance problems, misrepresenting operational information, misleading customers or other stakeholders, violating internal rules, or using improper means to protect organizational interests into their ethical risk monitoring systems. Anonymous reporting channels, periodic compliance surveys, and regular ethical risk reviews can help organizations identify potential unethical pro-organizational behavior. Particularly as employees’ ownership stakes increase, organizations should avoid evaluating ownership incentives solely on the basis of performance outcomes and should also consider whether the means used to achieve those outcomes comply with ethical and procedural standards. In this way, organizations can preserve the overall benefits associated with employee ownership while preventing potential ethical risks from being obscured by favorable performance outcomes.

Second, organizations should reinforce the responsibility-related meaning of employee ownership rather than emphasizing its economic returns alone. As ownership levels increase, the connection between employees’ personal economic interests and organizational performance becomes stronger. Organizations therefore have greater reason to clarify, through ownership-related briefings, employee training, and internal communication, that being an employee-owner entails not only sharing in organizational gains but also assuming responsibility for the processes through which those gains are achieved, the means used, and their longer-term consequences. Consistent with this principle, performance management and reward systems should not rely exclusively on financial indicators such as revenue, profit, market share, or stock value. They should also incorporate compliance, procedural integrity, stakeholder consequences, and other nonfinancial common-good indicators. Managers should likewise avoid messages implying that organizational goals must be achieved regardless of the means used, because such communication may further reinforce an exclusive focus on bottom-line outcomes. By evaluating economic outcomes alongside responsibility, compliance, and stakeholder-related consequences, organizations may reduce the likelihood that stronger economic stakes associated with higher ownership levels develop into a narrow bottom-line mentality.

Third, organizations should strengthen the alignment between the management of employee stock ownership levels, the broader HRM system, and formal governance mechanisms. The economic incentives associated with employee ownership should not operate independently of training, performance appraisal, reward systems, employee participation, and compliance governance. For example, organizations can combine financial indicators with nonfinancial common-good metrics in performance appraisal so that employees’ economic rewards depend not only on short-term performance but also on compliance, stakeholder responsibility, and long-term value creation. With respect to equity arrangements themselves, organizations may also consider longer or staggered vesting schedules that tie the economic value associated with higher ownership levels more closely to longer-term organizational outcomes and reduce excessive emphasis on short-term bottom-line results. In addition, firms can establish formal clawback provisions under which equity-related returns are forfeited, reduced, or recovered when performance is achieved through compliance violations, unethical pro-organizational behavior, or other practices that harm stakeholders. More importantly, organizations should periodically assess whether different management practices convey consistent goals and behavioral expectations. Even when employees hold relatively high levels of company stock, the stronger economic connection created by ownership does not necessarily translate into greater bottom-line mentality if performance and reward systems simultaneously emphasize the common good, compliance responsibilities, and long-term value creation. A more coherent combination of CGHRM practices, equity arrangements, and governance mechanisms may therefore help organizations better manage both the responsibility-related opportunities and the ethical risks associated with different levels of employee stock ownership.

### Limitations and future research

Despite the theoretical and practical contributions of this study, several limitations warrant consideration and provide opportunities for future research. First, although we adopted a three-wave, multisource matched survey design and reduced concerns about common method bias to some extent through temporal separation and the use of different data sources (Podsakoff et al., 2003), a time-lagged observational design does not establish strict causal relationships. The temporal ordering of the variables cannot completely rule out reverse relationships. In addition, felt responsibility and bottom-line mentality were both measured at Wave 2. Because we theorized them as parallel psychological pathways, this measurement arrangement is consistent with the current model, but it limits our ability to examine how these psychological states may evolve over time. Future research could employ longitudinal designs with longer time spans and additional measurement waves, or combine vignette experiments, field experiments, quasi-experimental designs, and cross-lagged panel models to obtain stronger causal evidence and further examine potential dynamic relationships among the focal variables.

Second, our data were collected from three firms in China, and the generalizability of our findings may therefore depend on both national- and firm-level contexts. Countries differ in their legal systems, capital markets, employment relations, and regulation of employee ownership, which may shape the economic and decision-making rights granted to employee-owners and how organizations define the employee-owner role (Caramelli and Briole, 2007). At the same time, even within the same national context, employee ownership arrangements may differ across firms in terms of equity instruments, vesting horizons, lock-up periods, restrictions on share transfers, and liquidity conditions. Such institutional features may affect the short-term economic incentives, longer-term interests, and responsibilities associated with different levels of employee ownership. Future research could therefore replicate our model across countries, industries, and firms while explicitly incorporating these design features. Doing so would help clarify the extent to which the ethical consequences associated with employee stock ownership levels depend on national institutional environments and firm-specific ownership arrangements, and would further identify the conditions under which the dual pathways identified in this study are more generalizable versus context dependent (Rousseau and Fried, 2001).

Third, the operationalization and sample scope of employee stock ownership also establish boundaries for the present findings. Building on prior employee ownership research (French and Rosenstein, 1984; Klein, 1987; Buchele et al., 2010), we measured employee stock ownership level using the absolute monetary value of company shares actually held by employees, which directly captures employees’ economic stake in the organization. However, the economic significance of a given ownership stake may vary depending on employees’ compensation, personal wealth, and hierarchical position. Moreover, these factors, along with prior performance, may be associated with both ownership levels and the psychological and behavioral outcomes examined in our study, raising potential endogeneity concerns. Although we controlled for functional department, including a senior-management category, compensation and prior performance were not directly controlled. Future research could incorporate relative measures of ownership and more comprehensive controls to further examine the robustness of our findings. In addition, because our study focused exclusively on differences in ownership levels among existing employee-owners, future research could include non-owner employees to distinguish the psychological and ethical consequences associated with ownership participation from those associated with differences in ownership level.

Fourth, drawing on goal-framing theory, we focused on felt responsibility and bottom-line mentality as two opposing psychological pathways. Although this theoretical perspective provides an integrative account of why employee stock ownership levels may be associated with divergent ethical consequences, felt responsibility and bottom-line mentality are unlikely to exhaust the potential mechanisms linking ownership levels to employee ethical behavior. Prior research suggests that employee ownership may also be associated with psychological processes such as psychological ownership, organizational identification, and employees’ attention to their personal economic interests (Dasilas, 2024; McConville et al., 2016; O’Boyle et al., 2016; Pierce et al., 1991). These mechanisms may operate independently of, or jointly with, the two pathways examined in our study to shape employees’ ethical judgments and behavior. For example, higher ownership levels may strengthen employees’ motivation to protect organizational interests through greater psychological ownership or organizational identification, while the tighter connection between ownership and personal economic returns may also affect employees’ willingness to cross ethical boundaries through other self-interested or instrumental motives. Future research could therefore draw on alternative theoretical perspectives to examine additional cognitive, affective, and motivational mechanisms associated with employee stock ownership levels and compare their relative explanatory roles, thereby developing a more comprehensive understanding of the potentially double-edged ethical consequences of employee ownership.

## Data Availability

The raw data supporting the conclusions of this article will be made available by the authors, without undue reservation.

## References

[ref1] Arain G. A. Hameed I. Crawshaw J. R. (2019). Servant leadership and follower voice: the roles of follower felt responsibility for constructive change and avoidance-approach motivation. Eur. J. Work Organ. Psychol. 28, 555–565. doi: 10.1080/1359432x.2019.1609946

[ref2] Aubert N. Garnotel G. Lapied A. Rousseau P. (2014). Employee ownership: a theoretical and empirical investigation of management entrenchment vs. reward management. Econ. Model. 40, 423–434. doi: 10.1016/j.econmod.2013.12.011

[ref3] Aust I. Matthews B. Müller-Camen M. (2020). Common good HRM: a paradigm shift in sustainable HRM? Hum. Resour. Manag. Rev. 30:100705. doi: 10.1016/j.hrmr.2019.100705

[ref4] Babalola M. T. Mawritz M. B. Greenbaum R. L. Ren S. Garba O. A. (2021). Whatever it takes: how and when supervisor bottom-line mentality motivates employee contributions in the workplace. J. Manag. 47, 1134–1154. doi: 10.1177/0149206320902521

[ref5] Berry C. M. Carpenter N. C. Barratt C. L. (2012). Do other-reports of counterproductive work behavior provide an incremental contribution over self-reports? A meta-analytic comparison. J. Appl. Psychol. 97, 613–636. doi: 10.1037/a0026739, 22201245

[ref6] Bliese P. D. (2000). “Within-group agreement, non-independence, and reliability: implications for data aggregation and analysis,” in Multilevel Theory, Research, and Methods in Organizations: Foundations, Extensions, and new Directions, eds. Klein K. J. Kozlowski S. W. J. (San Francisco, CA: Jossey-Bass), 349–381.

[ref7] Brislin R. W. (1980). “Translation and content analysis of oral and written materials,” in Handbook of cross-Cultural Psychology, eds. Triandis H. C. Berry J. W., vol. 2 (Boston: Allyn & Bacon), 389–444.

[ref8] Brown T. A. (2015). Confirmatory Factor Analysis for Applied Research. 2nd Edn. New York, NY: Guilford Press.

[ref9] Buchele R. Kruse D. L. Rodgers L. Scharf A. (2010). “Show me the money: does shared capitalism share the wealth?” in Shared Capitalism at Work: Employee Ownership, Profit and Gain Sharing, and Broad-based Stock Options, eds. Kruse D. L. Freeman R. B. Blasi J. R. (Chicago, IL: University of Chicago Press), 351–376.

[ref10] Caramelli M. Briole A. (2007). Employee stock ownership and job attitudes: does culture matter? Hum. Resour. Manage. Rev. 17, 290–304. doi: 10.1016/j.hrmr.2007.07.002

[ref11] Chen M. Chen C. C. Sheldon O. J. (2016). Relaxing moral reasoning to win: how organizational identification relates to unethical pro-organizational behavior. J. Appl. Psychol. 101, 1082–1096. doi: 10.1037/apl0000111, 27100068

[ref12] Dasilas A. (2024). The nonlinear relationship between employee stock ownership plans and firm performance: evidence from China. J. Bus. Res. 173:114470. doi: 10.1016/j.jbusres.2023.114470

[ref13] Farasat M. Azam A. (2022). Supervisor bottom-line mentality and subordinates’ unethical pro-organizational behavior. Pers. Rev. 51, 353–376. doi: 10.1108/pr-03-2020-0129

[ref14] Fehr R. Heng Y. T. Wang Y. Guo Y. (2025). They do not deserve your thanks! Witness reactions to leader-directed expressions of gratitude. J. Appl. Psychol. 110, 197–219. doi: 10.1037/apl0001228, 39146043

[ref15] Fox S. Spector P. E. Goh A. Bruursema K. (2007). Does your coworker know what you’re doing? Convergence of self- and peer-reports of counterproductive work behavior. Int. J. Stress. Manag. 14, 41–60. doi: 10.1037/1072-5245.14.1.41

[ref16] French J. L. Rosenstein J. (1984). Employee ownership, work attitudes, and power relationships. Acad. Manag. J. 27, 861–869. doi: 10.2307/255883, 14997687

[ref17] Fuller J. B. Marler L. E. Hester K. (2006). Promoting felt responsibility for constructive change and proactive behavior: exploring aspects of an elaborated model of work design. J. Organ. Behav. 27, 1089–1120. doi: 10.1002/job.408

[ref18] Greenbaum R. L. Mawritz M. B. Eissa G. (2012). Bottom-line mentality as an antecedent of social undermining and the moderating roles of core self-evaluations and conscientiousness. J. Appl. Psychol. 97, 343–359. doi: 10.1037/a0025217, 22268488

[ref19] Greenbaum R. L. Mawritz M. B. Zaman N. N. (2023). The construct of bottom-line mentality: where we’ve been and where we’re going. J. Manag. 49, 2109–2147. doi: 10.1177/01492063231153135

[ref20] Henseler J. Ringle C. M. Sarstedt M. (2015). A new criterion for assessing discriminant validity in variance-based structural equation modeling. J. Acad. Mark. Sci. 43, 115–135. doi: 10.1007/s11747-014-0403-8

[ref21] Kim A. Han K. (2019). All for one and one for all: a mechanism through which broad-based employee stock ownership and employee-perceived involvement practice create a productive workforce. Hum. Resour. Manag. 58, 571–584. doi: 10.1002/hrm.21958

[ref22] Kim E. H. Ouimet P. (2014). Broad-based employee stock ownership: motives and outcomes. J. Finance 69, 1273–1319. doi: 10.1111/jofi.12150

[ref23] Kim K. Y. Patel P. C. Devaraj S. (2025). Sorting, incentive, and investment effects of employee stock ownership. J. Bus. Res. 191:115277. doi: 10.1016/j.jbusres.2025.115277

[ref24] Klein K. J. (1987). Employee stock ownership and employee attitudes: a test of three models. J. Appl. Psychol. 72, 319–332. doi: 10.1037/0021-9010.72.2.319, 3583982

[ref25] Kline R. B. (2016). Principles and Practice of Structural Equation Modeling. 4th Edn. New York, NY: Guilford Press.

[ref26] LeBreton J. M. Senter J. L. (2008). Answers to 20 questions about interrater reliability and interrater agreement. Organ. Res. Methods 11, 815–852. doi: 10.1177/1094428106296642

[ref27] Lindenberg S. (2001). “Social rationality versus rational egoism,” in Handbook of Sociological Theory, ed. Turner J. H. (New York: Kluwer Academic/Plenum Publishers), 635–668.

[ref28] Lindenberg S. Foss N. J. (2011). Managing joint production motivation: the role of goal framing and governance mechanisms. Acad. Manag. Rev. 36, 500–525. doi: 10.5465/amr.2011.61031808

[ref29] Lindenberg S. Steg L. (2007). Normative, gain and hedonic goal frames guiding environmental behavior. J. Soc. Issues 63, 117–137. doi: 10.1111/j.1540-4560.2007.00499.x

[ref30] Long R. J. (1978). The effects of employee ownership on organizational identification, employee job attitudes, and organizational performance: a tentative framework and empirical findings. Hum. Relations 31, 29–48. doi: 10.1177/001872677803100102

[ref31] Lu Y. Zhang M. M. Yang M. M. Li T. (2025). Enhancing employee outcomes through common good human resource management: exploring the role of meaningfulness and thriving. Hum. Resour. Manag. 64, 485–502. doi: 10.1002/hrm.22270

[ref32] Lu Y. Zhang M. M. Yang M. M. Wang Y. (2023). Sustainable human resource management practices, employee resilience, and employee outcomes: toward common good values. Hum. Resour. Manag. 62, 331–353. doi: 10.1002/hrm.22153

[ref33] Mawritz M. B. Farro A. C. Kim J. Greenbaum R. L. Wang C. S. Bonner J. M. (2024a). Bottom-line mentality from a goal-shielding perspective: does bottom-line mentality explain the link between rewards and pro-self unethical behavior? Hum. Relat. 77, 505–532. doi: 10.1177/00187267221138187

[ref34] Mawritz M. B. Greenbaum R. L. Deng Y. Rosikiewicz B. L. Farro A. C. Mitchell M. (2024b). When competitive rewards create obsessions with bottom-line outcomes: a social interdependence theory perspective of the mediating role of bottom-line mentality. J. Organ. Behav. 45, 1231–1248. doi: 10.1002/job.2791

[ref35] McConville D. Arnold J. Smith A. (2016). Employee share ownership, psychological ownership, and work attitudes and behaviours: a phenomenological analysis. J. Occup. Organ. Psychol. 89, 634–655. doi: 10.1111/joop.12146

[ref36] Mo S. Lupoli M. J. Newman A. Umphress E. E. (2023). Good intentions, bad behavior: a review and synthesis of the literature on unethical prosocial behavior (UPB) at work. J. Organ. Behav. 44, 335–354. doi: 10.1002/job.2617

[ref37] Muthén L. K. Muthén B. O. (1998–2017). Mplus User’s Guide. 8th Edn Los Angeles, CA: Muthén & Muthén.

[ref38] O’Boyle E. H. Patel P. C. Gonzalez-Mulé E. (2016). Employee ownership and firm performance: a meta-analysis. Hum. Resour. Manag. J. 26, 425–448. doi: 10.1111/1748-8583.12115

[ref39] O’Brien R. M. (2007). A caution regarding rules of thumb for variance inflation factors. Qual. Quant. 41, 673–690. doi: 10.1007/s11135-006-9018-6

[ref40] Pearce J. L. Gregersen H. B. (1991). Task interdependence and extrarole behavior: a test of the mediating effects of felt responsibility. J. Appl. Psychol. 76, 838–844. doi: 10.1037/0021-9010.76.6.838

[ref41] Pendleton A. Robinson A. (2010). Employee stock ownership, involvement, and productivity: an interaction-based approach. ILR Rev. 64, 3–29. doi: 10.1177/001979391006400101

[ref42] Pendleton A. Robinson A. (2023). Employee behavior in employee stock option plans: why do some employees acquire company stock? Hum. Resour. Manag. 62, 197–211. doi: 10.1002/hrm.22139

[ref43] Pham N. T. Jabbour C. J. C. Pereira V. Usman M. Ali M. Vo-Thanh T. (2023). Common good human resource management, ethical employee behaviors, and organizational citizenship behaviors toward the individual. Hum. Resour. Manag. J. 33, 977–1000. doi: 10.1111/1748-8583.12493

[ref44] Pierce J. L. Rubenfeld S. A. Morgan S. (1991). Employee ownership: a conceptual model of process and effects. Acad. Manag. Rev. 16, 121–144. doi: 10.2307/258609

[ref45] Podsakoff P. M. MacKenzie S. B. Lee J. Y. Podsakoff N. P. (2003). Common method biases in behavioral research: a critical review of the literature and recommended remedies. J. Appl. Psychol. 88, 879–903. doi: 10.1037/0021-9010.88.5.879, 14516251

[ref46] Podsakoff P. M. MacKenzie S. B. Podsakoff N. P. (2012). Sources of method bias in social science research and recommendations on how to control it. Annu. Rev. Psychol. 63, 539–569. doi: 10.1146/annurev-psych-120710-100452, 21838546

[ref47] Podsakoff P. M. Organ D. W. (1986). Self-reports in organizational research: problems and prospects. J. Manag. 12, 531–544. doi: 10.1177/014920638601200408

[ref48] Rodríguez-Oramas A. Burgues-Freitas A. Joanpere M. Flecha R. (2022). Participation and organizational commitment in the Mondragon group. Front. Psychol. 13:806442. doi: 10.3389/fpsyg.2022.806442, 35369244 PMC8964931

[ref49] Rousseau D. M. Fried Y. (2001). Location, location, location: contextualizing organizational research. J. Organ. Behav. 22, 1–13. doi: 10.1002/job.78

[ref50] Steele L. M. Rees R. Berry C. M. (2024). The role of self-interest in unethical pro-organizational behavior: a nomological network meta-analysis. J. Appl. Psychol. 109, 362–385. doi: 10.1037/apl0001139, 37843545

[ref51] Umphress E. E. Bingham J. B. (2011). When employees do bad things for good reasons: examining unethical pro-organizational behaviors. Organ. Sci. 22, 621–640. doi: 10.1287/orsc.1100.0559, 19642375

[ref52] Umphress E. E. Bingham J. B. Mitchell M. S. (2010). Unethical behavior in the name of the company: the moderating effect of organizational identification and positive reciprocity beliefs on unethical pro-organizational behavior. J. Appl. Psychol. 95, 769–780. doi: 10.1037/a0019214, 20604596

[ref53] Wang J. Shi W. Liu G. Zhou L. (2021). Moving beyond initiative: the reconceptualization and measurement of unethical pro-organizational behavior. Front. Psychol. 12:640107. doi: 10.3389/fpsyg.2021.640107, 34658988 PMC8511457

[ref54] Weissenböck E. Breugst N. Patzelt H. Dibbern R. (2024). Equity ownership and identification with the founding team. Appl. Psychol. 73, 1626–1651. doi: 10.1111/apps.12515

[ref55] Xu L. Wen T. Wang J. (2022). How does job insecurity cause unethical pro-organizational behavior? The mediating role of impression management motivation and the moderating role of organizational identification. Front. Psychol. 13:941650. doi: 10.3389/fpsyg.2022.941650, 36211942 PMC9537742

[ref56] Yang X. Tseng Y. Lee B. (2021). Merging the social influence theory and the goal-framing theory to understand consumers’ green purchasing behavior: does the level of sensitivity to climate change really matter? Front. Psychol. 12:766754. doi: 10.3389/fpsyg.2021.766754, 34790155 PMC8591022

[ref57] Yin Y. Wang Y. Lu Y. (2025). How to design green compensation to promote managers’ pro-environmental behavior? A goal-framing perspective. J. Bus. Ethics 197, 341–353. doi: 10.1007/s10551-024-05762-4

[ref58] Zhang Y. He B. Huang Q. Xie J. (2020). Effects of supervisor bottom-line mentality on subordinate unethical pro-organizational behavior. J. Manag. Psychol. 35, 419–434. doi: 10.1108/jmp-11-2018-0492

